# Reassessment of hydrogen tolerance in *Caldicellulosiruptor saccharolyticus*

**DOI:** 10.1186/1475-2859-10-111

**Published:** 2011-12-21

**Authors:** Karin Willquist, Sudhanshu S Pawar, Ed WJ Van Niel

**Affiliations:** 1 Department of Applied Microbiology, Lund University, P.O. Box 124, SE-221 00 Lund, Sweden

**Keywords:** *Caldicellulosiruptor saccharolyticus*, biohydrogen production, hydrogen tolerance, enzyme levels, glyceraldehyde-3-phosphate dehydrogenase kinetics, redox ratio

## Abstract

**Background:**

*Caldicellulosiruptor saccharolyticus *has the ability to produce hydrogen (H_2_) at high yields from a wide spectrum of carbon sources, and has therefore gained industrial interest. For a cost-effective biohydrogen process, the ability of an organism to tolerate high partial pressures of H_2 _(*P*_H2_) is a critical aspect to eliminate the need for continuous stripping of the produced H_2 _from the bioreactor.

**Results:**

Herein, we demonstrate that, under given conditions, growth and H_2 _production in *C. saccharolyticus *can be sustained at *P*_H2 _up to 67 kPa in a chemostat. At this *P*_H2_, 38% and 16% of the pyruvate flux was redirected to lactate and ethanol, respectively, to maintain a relatively low cytosolic NADH/NAD ratio (0.12 mol/mol). To investigate the effect of the redox ratio on the glycolytic flux, a kinetic model describing the activity of the key glycolytic enzyme, glyceraldehyde-3-phosphate dehydrogenase (GAPDH), was developed. Indeed, at NADH/NAD ratios of 0.12 mol/mol (*K*i of NADH = 0.03 ± 0.01 mM) GAPDH activity was inhibited by only 50% allowing still a high glycolytic flux (3.2 ± 0.4 mM/h). Even at high NADH/NAD ratios up to 1 mol/mol the enzyme was not completely inhibited. During batch cultivations, hydrogen tolerance of *C. saccharolyticus *was dependent on the growth phase of the organism as well as the carbon and energy source used. The obtained results were analyzed, based on thermodynamic and enzyme kinetic considerations, to gain insight in the mechanism underlying the unique ability of *C. saccharolyticus *to grow and produce H_2 _under relatively high *P*_H2_.

**Conclusion:**

*C. saccharolyticus *is able to grow and produce hydrogen at high *P*_H2_, hence eliminating the need of gas sparging in its cultures. Under this condition, it has a unique ability to fine tune its metabolism by maintaining the glycolytic flux through regulating GAPDH activity and redistribution of pyruvate flux. Concerning the later, xylose-rich feedstock should be preferred over the sucrose-rich one for better H_2 _yield.

## Background

In the continuous quest for an economically competitive biohydrogen production plant, it is important to obtain as high H_2 _yields as possible [1]. The H_2 _yields reported in literature for dark fermentation with various mesophilic microorganisms are usually in the range of 1-2 moles per mole of hexose [2-5], whereas a maximum empirical yield can be gained of 4 mol H_2_/mol hexose [6]. One successful strategy to maximize H_2 _yields is to carry out the fermentation with (hyper)thermophiles at elevated temperatures. This makes the H_2_-generation reactions more energetically favourable [7] and has, therefore, a positive impact on H_2 _yields [5,8]. Indeed, the highest H_2 _yields reported to date, approaching the theoretical maximum, were obtained with (hyper)thermophiles [9-11].

Since H_2 _is known to have an inhibitory effect on growth and its own production in a variety of microorganisms, including (hyper)thermophiles [5,8], maximizing fermentative H_2 _yield is made possible by keeping the *P*_H2 _in the fermentation vessel sufficiently low. Normally, this is ensured by continuous stripping of the H_2 _from the production broth using an inert gas, such as N_2 _or He [12]. However, using an inert gas requires a subsequent energy-demanding gas-upgrading step [13]. Instead, CO_2 _might offer an economic alternative [14] as it is a by-product of the fermentation process and can be more readily separated from H_2 _[13]. However, stripping with CO_2 _increases the osmolality of the fermentation broth, ultimately reducing the growth of the H_2_-producing organism[15]. To avoid stripping, high H_2 _yields have simply to be obtained at high *P*_H2_.

*Caldicellulosiruptor saccharolyticus *is a strict anaerobic, extreme thermophilic bacterium that is able to produce nearly stoichiometric amounts of H_2 _from glucose [9] and sucrose [16]. In addition, *C. saccharolyticus *has the unique ability to co-metabolize a wide spectrum of carbohydrates including both pentoses and hexoses [17,18], and to break down complex hemi-cellulosic materials as well as other complex polysaccharides [19-22]. The genome of this organism has been recently sequenced [17] facilitating improved discernments of its metabolic network.

High H_2 _yields can only be achieved when acetate is the main metabolic by-product, since the formation of more reduced products, such as lactate and ethanol, drains electrons from H_2 _production. In this work, we evaluated the influence of elevated *P*_H2 _on growth, the extent of lactate formation and accordingly H_2 _yields, by *C. saccharolyticus *in batch as well as carbon-limited continuous cultures, with glucose as the main carbon and energy source. The effect of *P*_H2 _on *C. saccharolyticus *metabolism on pentoses (xylose) was also evaluated in batch cultures and was compared with previous results on disaccharides (sucrose; [23]). We demonstrate that, depending on the growth conditions, the organism can grow and produce H_2 _at *P*_H2 _up to 67 kPa. The activity levels of three redox-related catabolic enzymes were compared in the presence and the absence of N_2 _sparging and correlated with product distribution under each condition. In addition, a kinetic model was developed to examine the influence of the changes in the intracellular levels of NADH on the activity of a key glycolytic enzyme, glyceraldehyde-3-phosphate dehydrogenase (GAPDH), and is compared with other related organisms. The obtained results are analyzed, based on thermodynamic considerations, to understand the mechanism underlying the unique ability of *C. saccharolyticus *to grow and produce H_2 _under relatively high *P*_H2_.

## Materials and methods

### Microorganism and culture medium

*C. saccharolyticus *DSM 8903 was purchased from the Deutsche Sammlung von Mikroorganismen und Zellkulturen (Braunschweig, Germany). A modified DSM 640 medium [15] was used for all cultivations throughout this work. Routine subcultures and inoculum development were conducted in 250-mL serum bottles containing 50-mL of medium. Anoxic solutions of different carbon sources were autoclaved separately and added to the sterile medium at the required concentration.

### Fermentation setup

Cultures were grown in a jacketed, 3-L bioreactor equipped with an ADI 1025 Bio-Console and an ADI 1010 Bio-Controller (Applikon, Schiedam, The Netherlands) at a working volume of 1L, either in batch or continuous mode. The pH was maintained at 6.5 ± 0.1 at 70°C by automatic titration with 4 M NaOH. The temperature was thermostatically kept at 70 ± 1°C and the stirring rate was set to 250 rpm. A condenser with 5°C cooling water was fitted to the bioreactor's headplate. Prior to inoculation, the medium was sparged with N_2 _and supplemented with an anoxic solution of cysteine-HCl at a final concentration of 1 g L^-1 ^to render the medium completely anaerobic. For continuous cultivations, the bioreactor was started to be fed with fresh medium at the end of the logarithmic growth phase of the culture, having an identical composition to the batch start-up medium, except for cysteine-HCl (final concentration of 0.25 g/L in medium bottle) at the required dilution rate (*D*). Steady states were assessed after at least 5 volume changes based on the criteria of constant H_2 _and CO_2 _production rates and constant biomass concentration. Glucose was used as a primary substrate in all batch and continuous experiments at an initial concentration of 5 g/L, if not stated otherwise.

Three different experimental designs were applied: continuous flushing with 100 mL min^-1 ^N_2 _for continuous removal of produced H_2 _(*Case I*); no gas sparging, with the bioreactor's gas outlet open leading to higher concentrations of H_2 _in the headspace at 1 bar (*Case II*); no gas sparging, with the bioreactor's gas outlet closed allowing H_2 _to accumulate and increasing the total pressure in the bioreactor (*Case III*). Gas samples from the headspace for H_2 _and CO_2 _determination and culture samples for monitoring growth, substrate consumption and product formation were regularly withdrawn during fermentation. At steady states, samples were taken for determining the NADH/NAD ratio and cell dry weight (CDW) and anaerobic culture samples for enzyme activity measurements as described previously [24]. Continuous cultivations were performed at the dilution rates of 0.05 h^-1 ^and 0.15 h^-1 ^in duplicate under both, '*Case I*' and '*Case II*', conditions.

### Analytical methods

Headspace samples were analyzed for CO_2 _and H_2 _by gas chromatography, using a dual channel Micro-GC (CP-4900; Varian, Micro gas chromatography, Middelburg, The Netherlands), as previously described [11]. The results were analyzed with a Galaxie Chromatography Workstation (v.1.9.3.2). The optical density of the culture was measured at 620 nm (OD_620_) using a U-1100 spectrophotometer (Hitachi, Tokyo, Japan). CDW was determined by filtration as previously described [24]. Glucose, acetate, lactate, succinate and ethanol were analyzed by HPLC (Waters, Milford, MA, USA) on an Aminex HPX-87H ion exchange column (Bio-Rad, Hercules, USA) at 45°C, with 5 mM H_2_SO_4 _(0.6 ml min^-1^) as the mobile phase. The column was equipped with a refractive index detector (RID-6A; Shimadzu, Kyoto, Japan).

### Preparation of cell extracts

Cell extracts (CE) were prepared anaerobically in duplicates using cells harvested from continuous cultures. All cell manipulations were carried out in an anaerobic glove box (Plas Labs Inc., MI, USA) with a N_2_/H_2_/CO_2 _atmosphere (85/10/5 v/v). Cell suspensions were centrifuged outside the glove box for 5 min at 5,000 × *g *and 4°C, after the addition of sodium dithionite at a final concentration of 5.2 mg L^-1 ^to ensure complete anaerobiosis. The cell pellets were resuspended in a reaction buffer (0.1 M Tris-HCl containing 40 mM NaCl and 5 mM MgCl_2_; pH 7.2) [24] Cells were mixed with an equal volume of 0.1 mm silica beads and disrupted in a Mini-Beadbeater (BioSpec Products Inc., OK, USA) in 3 cycles of 20 s beating and 60 s cooling. Cell debris was removed by 5 min centrifugation at 12,000 × *g *(Minispin, Eppendorf, Hamburg, Germany) and the resulting CE was either used directly or stored under anaerobic conditions at -20°C until use. For determination of GAPDH kinetics, the CE was freed from compounds with a M_W _below 5 kDa using a PD10 column (Sigma-Aldrich), as previously described [24].

### Enzyme assays

All enzyme activity measurements were carried out as described previously [24], with the modification that 5.35 mM GAP was used in the assay for GAPDH activity. All assays were carried out in at least three technical replicates in the linear protein concentration range. In addition, the influence of the metabolites ATP, ADP and PPi on GAPDH activity in the concentration range of 1-10 mM was evaluated. The *K*_0.5 _for the substrates GAP and NAD^+ ^were determined by using seven different GAP concentrations and varying the NAD^+ ^concentration. The *Ki *for NADH was determined by using four different NAD^+ ^concentrations and varying NADH concentration.

Background reactions for the assays were determined by replacing the substrate with the reaction buffer. One unit of enzyme activity (IU) is defined as the amount of enzyme that catalyzes the conversion of 1 μmol of substrate per min. Specific activities are expressed as IU (mg protein)^-1^. Protein concentration in the CE was determined according to Bradford (1976), with bovine serum albumin as a standard.

### NAD(H)assay

The intracellular concentrations of NADH and NAD were determined by a cyclic assay as described earlier [25,24], with the exception of using phenazine ethosulfate (PES) instead of phenazine methosulfate (PMS), as PES is chemically more stable than PMS [25].

### Measurement of ATP and PPi

Samples were collected in screw-cap microcentrifuge tubes containing ice-cold chloroform and immediately frozen into liquid nitrogen. Samples were stored at -80°C until further analysis. During the sample preparation cells were not separated from the medium to avoid the loss of ATP and/or PPi due to possible leakage during centrifugation, as previously observed by Bielen et al [26]. Moreover, assays were also done to estimate the levels of ATP and PPi in the growth medium. ATP and PPi were extracted from the cells by using the cell lysis buffer as described in the protocol provided with the ATP Bioluminescence assay kit HSII (Roche Molecular Biochemicals). ATP was measured using the ATP Bioluminescence assay kit HSII (Roche Molecular Biochemicals) containing luciferin/luciferase reagent, according to the protocol provided with the kit, in a tube-reading 1250 Luminometer (LKB-Wallac, Turku, Finland).

Samples for PPi measurement were treated with ATP-sulfurylase (Sigma-Aldrich, Germany) in the presence of excess Adenosine-5'-phosphosulfate to produce ATP from PPi [27] and the overall ATP was measured with the Bioluminescence assay kit HSII (Roche Molecular Biochemicals). Since significant amounts of ATP were present in the samples, the assay was started with the measurement of ATP, to convert most of the ATP into PPi and immediately ATP sulfurylase was added to convert overall PPi into ATP, which was subsequently measured. The background signal, less than 15% of the total signal in all measurements, was subtracted from the total signal to estimate the net PPi concentration. Intracellular levels of ATP and PPi were calculated as previously described [26].

### Calculations

H_2 _productivity (mM h^-1^) and cumulative H_2 _formation (CHF, mM) were calculated in two different ways depending on the experimental design. All calculations were based on the ideal gas law using H_2 _and CO_2 _concentration in the headspace. For *Case I *(sparging with N_2_) the calculations were based on the flow rate of the influent N_2 _gas and the percentages of H_2 _and CO_2 _in the effluent gas, as no other gases were detected, whereas for *Case II *(no sparging) the flow rate of the effluent gas was measured by the water displacement method with CO_2_-saturated water to avoid any further CO_2 _to dissolve. It was assumed that CO_2 _in the effluent gas did not dissolve in the CO_2_-saturated water; therefore the actual dissolved CO_2 _concentration was not determined. CO_2_-saturated water was prepared by stripping the boiling water with 100% CO_2_, cooling it down simultaneously and was kept cold throughout the experiment. At the steady state, the flow rate of the effluent gas was determined by measuring the volume of the effluent gas collected between two time points. Thus, H_2 _productivity and CHF were calculated based on hydrogen concentration in the effluent gas and the flow rate of the effluent gas.

The intracellular specific productivities (mmol.g^-1^.h^-1^), i.e. *q*_NADH _produced, *q*_NADH _used, *q*_NADH _available and *q*_pyruvate_, were estimated as described previously [28].

The biomass yield per mol of ATP (*Y*_x/ATP_; g.mol^-1^) was calculated based on the equation previously described [15]:

(1) $$Y_{\text{x/ATB}}=\frac{\left[\text{biomass}\right]}{1.5\times \left[\text{acetate}\right]+0.5\times \left[\text{lactate}\right]+0.5\times \left[\text{ethanol}\right]}$$

The dissolved H_2 _concentration (H_2,aq_) in equilibrium was estimated according to Henry's law:

(2) $${\text{H}}_{2,\text{aq}}=P_{\text{H}2}\times K_{\text{H}}$$

where *K*_H _is Henry's constant (mM/bar) and is dependent on the temperature according to:

(3) $$\ln\frac{K_{2}}{K_{1}}=\frac{\Delta H^{0}}{R}*\left[\frac{1}{T_{1}}-\frac{1}{T_{2}}\right]$$

where *K*_1 _is *K*_H _at *T*_1 _= 298 K (0.78 mM/bar, [29]), *K*_2 _is the calculated *K*_H _at *T*_2 _= 343 K (0.52 mM/bar), ΔH (J/mol) is the enthalphy at standard conditions and R (8.314 J/mol/K) is the gas constant.

### 2.9 GAPDH model, data fitting and statistical analysis

The affinity constants for the substrates GAP and NAD^+ ^was determined by fitting the Michaelis-Menten type kinetic equation to the obtained data [30]:

(4) $$v=V_{\max}*\frac{\left[\text{NA}{\text{D}}^{+}\right]*\left[\text{GAP}\right]}{K_{GAP}*\left[NAD^{+}\right]+K_{NAD}*\left[\text{GAP}\right]+\left[\text{GAP}\right]*\left[\text{NA}{\text{D}}^{+}\right]+\alpha *K_{GAP}}$$

where *V *is the reaction rate, *V*_max _is the maximum rate of the reaction, *K_GAP _*and *K_NAD _*are the affinity constants for GAP and NAD^+^, respectively, and *α *is a constant representing any interaction between NAD and GAP binding to the enzyme. However, from analysis of our data *α *was not significantly higher than zero and was, therefore, the term "α× *K_GAP_*" was excluded from the equation.

The inhibition kinetics of NADH was determined by using four different NAD^+ ^concentrations and varying NADH concentrations. The type of inhibition kinetics was visualized by fitting equations for i) sigmoidal competitive inhibition, ii) uncompetitive inhibition iii) mixed inhibition, or iv) linear competitive inhibition

(5) $$v=\frac{V_{\max}*\left(\text{NA}{\text{D}}^{+}\right)*\left(\text{GAP}\right)}{\left[K_{GAP}+\left(\text{GAP}\right)\right]*\left[K_{NAD}*\left(1+\left(\frac{NADH}{K_{NADH}}\right)\right)+\left(\text{NA}{\text{D}}^{+}\right)\right]}$$

where *K_NADH _*is an inhibition constant, to the experimental data [30].

Data obtained from the inhibition kinetics of NADH was also used to study the effect of NADH/NAD (redox ratio) on GAPDH activity by assuming redox ratio as a substrate. Hill type kinetic equation was fitted to the data [30]:

(6) $$v=\frac{V_{\max}}{1+{\left(\frac{K_{R}}{S^{}}\right)}^{h}}$$

where S is the redox ratio, *K_R _*is an affinity constant and *h *is the Hill coefficient of cooperatively.

Parameter estimation (viz. *V*_max_, α, *K_GAP_*, *K_NAD_*, *K_NADH_*, *K_R _*and *h*) was based on non-linear regression using the Surface Fitting Tool (sftool) or curve fitting tool (cftool) in MATLAB (R2009a), which also provides a statistical analysis. Model discrimination was based on the goodness of fit, which was evaluated by the 95% confidence bounds for the fitted parameters and by the square of the multiple correlation coefficients (*R*^2^).

Estimations on LDH activity was based on previously published model of LDH regulation in *C. saccharolyticus *[24].

## Results and discussion

### 3.1 Effect of P_H2 _on growth and lactate formation in batch cultures

**Figure 1:**
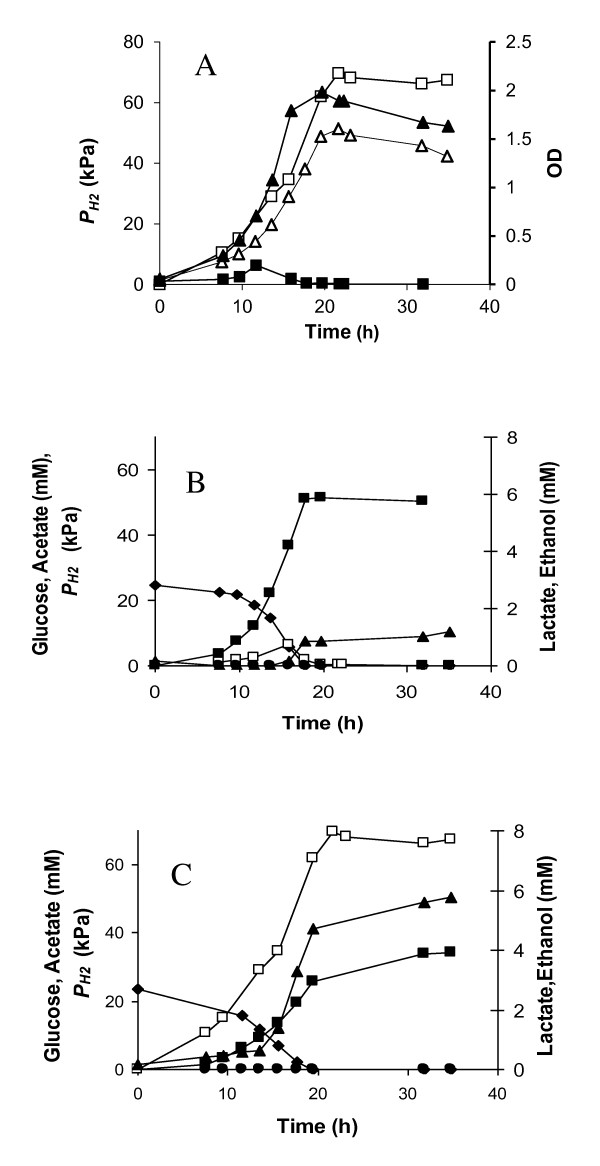
**Growth and product formation by *C. saccharolyticus *in pH-controlled batch fermentations with and without N_2 _sparging.** (A) with sparging (filled symbols) and without sparging (open symbols) growth (▲, Δ) and *P*_H2 _(■, □). Product formation in the presence (B) and absence of N_2 _sparging (C) glucose (♦), acetate (■), lactate (▲), ethanol (●) and *P*_H2 _(□). Note: Different scales are used on primary and secondary vertical axes.

*C. saccharolyticus *was cultivated in pH-controlled batch mode, with and without N_2 _sparging. The *P*_H2 _peaked at 6.3 kPa with sparging and 67 kPa without sparging the culture, which allowed analyzing the influence of the *P*_H2 _on growth and product formation. In both cases, the organism grew at a similar rate until a critical *P*_H2 _of 11 kPa was reached in the gas phase of the non-sparged fermentor, at which the growth rate decreased by 24% (Figure 1A). Moreover, when the *P*_H2 _reached 30 kPa (after 14 h of growth; Figure 1A) growth became linear. In contrast, the sparged culture grew exponentially until glucose was almost depleted after 16 hours of incubation (Figure 1A and 1B). Inhibition of growth is probably a result of both high dissolved hydrogen concentration and high osmolarity due to high dissolved CO_2 _concentrations in the non-stripped reactor [31].

Consistent with previous findings [24], acetate and H_2 _were the main metabolic end products during exponential growth when the culture was sparged with N_2 _(Figure 1B). Lactate formation was in this case initiated in the transition to stationary phase at a low *P*_H2 _(6.3 kPa).

Moreover, although some lactate was produced during early growth in the absence of sparging, the lactate productivity accelerated when the growth became linear after 14 hours of cultivation (Figure 1C). At this point, the *P*_H2 _was 30 kPa, thus about 2-fold higher than the previously quoted critical *P*_H2 _for lactate formation (10-20 kPa; [23]). The H_2 _productivity was not determined due to technical difficulties in accurate determination of instable increasing productivities with water displacement techniques. These results indicated that in batch cultivations, the cells can withstand higher *P*_H2 _maintaining an exponential growth profile until lactate is started to be formed, accompanied with linear growth.

The increased *P*_H2 _clearly influenced the overall lactate yield, as the final lactate concentration was 5-fold higher when the culture was not sparged with N_2 _(Figure 1B and 1C). The acetate/lactate ratio was 43 and 6 for the sparged and the non-sparged conditions, respectively. Ethanol, which acts as an alternative electron sink for *C. saccharolyticus*, was present in negligible quantities, irrespective of the *P*_H2 _(Figure 1B, C).

This behaviour is consistent with the outcome of another study on metabolic shifts in *C. saccharolyticus *[31], which demonstrated that a combination of the osmotic pressure and the dissolved H_2 _concentration determines the metabolic shift to lactate production. The key players behind this are the intracellular energy carriers that influence the lactate dehydrogenase (LDH) [24]. The kinetics of LDH demonstrated that the anabolic byproduct and energy carrier, PPi, plays a central role in the allosteric regulation of this catabolic enzyme by acting as a strong competitive inhibitor (*K*i = 1.7 mM), therefore antagonizing the stimulating effect of elevated NADH/NAD ratios [24]. The PPi levels in *C. saccharolyticus *are correlated to the growth rate, as the PPi concentration is highest (4 ± 2 mM) during exponential growth, and decreased seven folds during the transition to the stationary phase [26]. Consequently, at exponential growth, these high PPi levels assure that LDH remains inactive even at higher NADH/NAD (1.2 mol.mol^-1^) ratios. However, when the growth rate decelerates the concomitant decrease in PPi levels enables LDH to become sensitive to an increase in the NADH/NAD ratio [24]. Therefore, lactate is not formed even at high *P*_H2 _(< 30 kPa), as long as the cells are able to sustain high PPi levels through maintaining a high growth rate.

### Effect of P_H2 _on growth and lactate formation in continuous culture

Carbon-limited chemostat cultures were used to investigate the effect of *P*_H2 _on *C. saccharolyticus *at a controlled physiological state. In the absence of gas sparging, the *P*_H2 _reached 67 kPa after about 10 volume changes at *D *= 0.05 h^-1 ^without any washout of the culture indicating that *C. saccharolyticus *can withstand higher *P*_H2 _than was previously reported [23]. A noticeable effect of the elevated *P*_H2 _in this case was the redirection of the pyruvate flux, i.e. 38% and 16% of the flux at the pyruvate node was directed to lactate and ethanol, respectively, whereas 0.5% and 4.1% of corresponding fluxes were observed in sparged cultures at similar dilution rates (Table 1). Interestingly, the overall catabolic rate (q_glucose_) was not reduced at this high *P*_H2 _(Table 1). However, a steady state could not be attained in the absence of N_2 _sparging at a higher *D *(0.15 h^-1^) and the culture washed out at *P*_H2 _of 67 kPa. These results are in line with previous findings of supersaturation of hydrogen around the cell surface at high productivities due to mass transfer limitation [31]. Under the assumption of an equilibrium between dissolved (H_2,aq_) and gaseous H_2_, the H_2,aq _should be 0.24 μM at 67 kPa in the headspace and 70°C (Eq. 2 and 3), hence well below the critical H_2,aq _concentration for growth (H_2,aq crit _= 2.2 mM; [31]. However, the actual concentration around the cell depends on the ratio of H_2 _productivity/H_2 _mass transfer rate [31]. Therefore, the observation that the cells washed out at high D (0.15 h^-1^) but retained at low D (0.05 h^-1^) strongly indicates that at a lower growth rate, the H_2 _productivity is in the same range as compared to the mass transfer rate such that H_2,aq _< H_2,aq crit_. In contrast, at the higher growth rate, the hydrogen productivity exceeds the mass transfer rate by far, especially under non-sparging conditions, resulting in supersaturation of hydrogen and thus extensive growth inhibition [31].

**Table 1:** Fermentation data in continuous cultivations of *C.saccharolyticus *on glucose (5g.L^-1^) at steady states of different dilution rates, with and without N_2 _sparging.

Parameter	Results obtained with and without N_2 _sparging at *D *(h^-1^) of:		
	
	0.05 (100 mL/min N_2_)	0.15 (100 mL/min N_2_)	0.05 no stripping
Biomass conc. (g/L)	0.51 ± 0.02	0.61 ± 0.05	0.42 ± 0.01
Residual glucose conc. (mM)	0.05 ± 0.03	3 ± 2	0.3 ± 0.3
*q_glucose _*(mmol/g/h)	2.90 ± 0.09	6.2 ± 0.4	3.2 ± 0.4
*q_pyruvate_*(mmol/g/h)	4.90 ± 0.19	9.55 ± 0.03	5.8 ± 0.7
*q_H2 _*(mmol/g/h)	10. 1 ± 0.4	18.0 ± 0.0	5.9 ± 0.6
Product yield (mol/mol)			
H_2_	3.48 ± 0.09	2.9 ± 0.2	1.82 ± 0.03
Acetate	1.61 ± 0.03	1.32 ± 0.13	0.83 ± 0.02
Lactate	0.01 ± 0.00	0.01 ± 0.01	0.67 ± 0.01
Ethanol	0.07 ± 0.00	0.20 ± 0.03	0.28 ± 0.04
Biomass (g/mol)	17.3 ± 0.5	24.1 ± 1.5	16 ± 2
Y_ATP _(g cells/mol ATP)	5.4 ± 0.2	9.1 ± 0.3	6.7 ± 0.6
Carbon recovery (%)	0.96 ± 0.01	0.93 ± 0.04	0.93 ± 0.02
Redox recovery (%)	0.98 ± 0.01	0.93 ± 0.03	0.97 ± 0.02

Under N_2_-sparging conditions, the dilution rate also had an effect on product distribution. At *D *= 0.15 h^-1^, the H_2 _yield was lower than at *D *= 0.05 h^-1 ^(2.9 ± 0.2 mol/mol glucose and 3.48 ± 0.09 mol H_2_/mol glucose, respectively), which agrees with the findings of de Vrije et al [9]. Moreover, only 86% of pyruvate flux was directed to acetate at *D *= 0.15 h^-1^, compared to a 95% flux at *D *= 0.05 h^-1^. In addition, as previously reported [9], more residual glucose was observed at higher *D *(Table 1).

The biomass yield of *C. saccharolyticus *at low *P*_H2 _is slightly higher than that in *Clostridium cellulolyticum *[32], but similar to that in *Thermoanaerobacter ethanolicus *[33], and significantly lower than that in *Cl. acetobutylicum *[34]. The increase in the energetic biomass yield of *C. saccharolyticus *at the higher *D *was also observed for *Cl. cellulolyticum *[32] and *T. ethanolicus *[33].

### Level of key redox-related catabolic enzymes

The increase in *P*_H2 _appeared to enhance lactate formation in *C. saccharolyticus *(Table 1). Therefore, the activities of different catabolic dehydrogenase enzymes were determined in cells grown in continuous cultures (Table 2). The specific activity of GAPDH decreased about 60% with an increase in the growth rate. On the other hand, the opposite trend was observed in the specific activities of LDH and ADH. The level of specific LDH activity increased almost eight folds in absence of sparging, which corresponded well with the observed increase in the lactate flux (Table 1, 2). This correlation is comparable to the 2-fold increase in specific LDH activity and lactate flux during the transition to the stationary phase in batch cultures of *C. saccharolyticus *on 10 g.L^-1 ^glucose [24]. Similarly, ADH specific activity increased four folds in absence of sparging (Table 2) consistent with four-fold increase in the ethanol flux (Table 1). It has been shown previously that the levels of this enzyme in *C. saccharolyticus *increased three folds during batch growth at the onset of the stationary phase [24]. The levels of the GAPDH and ADH in *C. saccharolyticus *under N_2 _sparging were comparable to the measured levels of the corresponding enzymes in *Cl. cellulolyticum *under equivalent conditions [32]. However, LDH activity was about three folds higher in sparged cultures of *Cl. cellulolyticum *at the low dilution rate [32], which could be a consequence of the strong regulation of the enzyme [24].

**Table 2:** Enzyme levels (IU.(mg protein^-1^)) of key catabolic redox-dependent enzymes at steady states at different dilution rates, i.e. GAPDH, ADH, LDH and estimated activity (% of potential activity) of LDH (LDHv) under physiological conditions (Tables 3, 4) based on previously described kinetic model [23], of *C. saccharolyticus *cultures in the presence and absence of N_2 _sparging. Presented data is average of one biological and at least three technical replicates at a linear range.

Enzyme	Results obtained with and without N_2 _sparging at *D *(h^-1^) of:		
	
	0.05 (100 mL/min N_2_)	0.15 (100 mL/min N_2_)	0.05 no sparging
GAPDH	3.5	1.4	1.3
ADH	0.44	0.96	1.8
LDH	1.2	2.6	8.3
LDHv	10	25	100

### Inhibition of GAPDH by NADH

**Figure 2:**
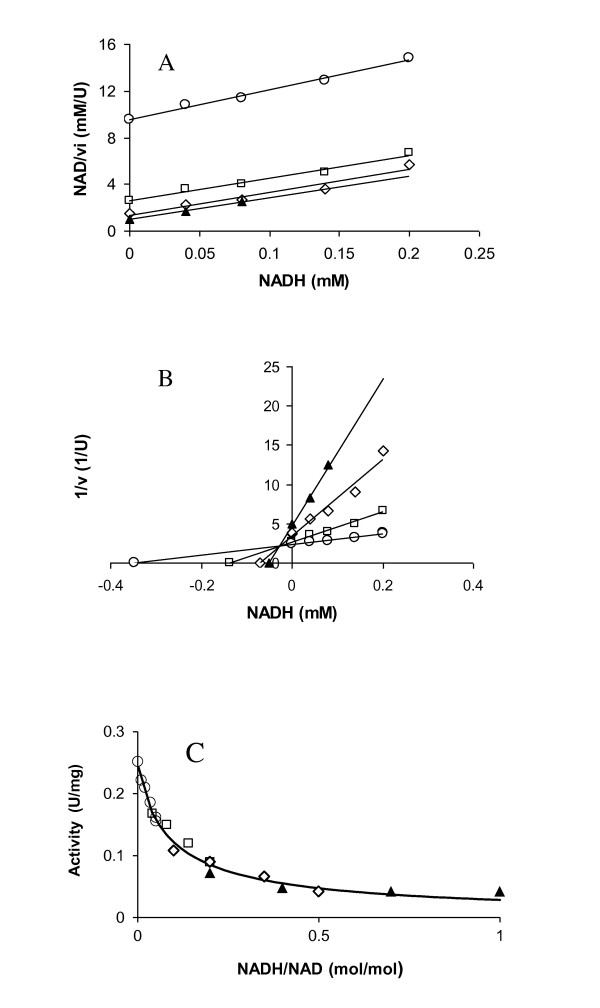
**Inhibition of GAPDH of *C. saccharolyticus *by NADH.** (A) Cornish-Bowden plot; (B) Dixon plot and (C) the effect of NADH/NAD ratio on specific activity of GAPDH with four different NAD concentrations; 0.2 mM (▲), 0.4 mM (◇), 1 mM (□) and 4 mM (○).

To investigate the effect of increased dissolved hydrogen concentration on the glycolytic flux, the effect of NAD and NADH on GAPDH activity in *C. saccharolyticus *was investigated *in vitro*. Conversion of GAP and NAD by GAPDH followed Michaelis-Menten kinetics, with *K*_0.5 _values of 1.5 ± 0.3 and 0.28 ± 0.06 mM for GAP and NAD, respectively. Fitting the kinetic model to the data (Eq. 6) showed that there is no interaction between NAD and GAP when binding to the enzyme. NADH inhibited the reaction in a competitive manner (*K*_NADH _= 0.03 ± 0.01 mM; Eq. 7), based on Dixon and Cornish-Bowden plots (Figure 2A and 2B) as well as through model discrimination by comparative fitting different inhibition models to the data (*R*^2 ^= 0.974 for the competitive model; data not shown). The activity of GAPDH appeared to follow Hill kinetics with respect to changes in NADH/NAD ratio (Figure 2C; *R*^2 ^= 0.9817; *K*_R _= 0.09 ± 0.01; *h *= -0.8 ± 0.1). Moreover, increase in the NADH/NAD ratio up to one, was unable to fully inhibit the enzyme (Figure 2C). Based on the estimated value for *K*i of NADH, GAPDH in *C. saccharolyticus *was more resistant to increased NADH levels than the GAPDH of most other related bacteria. The NADH concentration that causes 50% inhibition of the enzyme of *C. saccharolyticus *was 0.03 mM, as compared to 0.01 mM for *T. thermohydrosulfuricus *(formerly known as *Clostridium thermohydrosulfuricum*; [35]) and *Cl. acetobutylicum *[34]. However, the enzyme of *C. saccharolyticus *is less resistant to NADH inhibition than that of *Cl. cellulyticum *[36], for which 50% inhibition was observed at 0.1 mM NADH (Figure 2C). Consistently, the NADH/NAD ratio in the cells of *Cl. cellulyticum *is significantly higher than in *C. saccharolyticus *[24,37].

Finally, the activity of GAPDH was not found to be influenced by ATP, ADP or PPi over the physiological range of metabolite concentrations (1-10 mM).

### Redox metabolism and its effect on the glycolytic flux

High *P*_H2 _can potentially inhibit H_2 _formation through product inhibition of the hydrogenase-catalyzed NADH oxidation [38], which could lead to increased NADH/NAD ratios [37]. To investigate whether this was the case for *C. saccharolyticus *in the absence of sparging, NADH and NAD levels were determined at steady state conditions with and without sparging (Table 3). Interestingly, the NADH/NAD ratio remained similar (0.12 mol/mol; Table 3) at which the GAPDH activity was decreased for about 50% by NADH inhibition (Figure 2C). These results indicate that *C. saccharolyticus *sustains the NADH/NAD ratio at a homeostatic level as to support a high glycolytic flux. This could be achieved inside the cells by two mechanisms, i) by regulating the activity of GAPDH and/or ii) by redirecting pyruvate flux to more reduced products.

**Table 3:** Measured NADH/NAD ratios and NADH concentration and estimated redox fluxes at steady states of different dilution rates of *C.saccharolyticus *cultures in the presence and absence of N_2 _sparging

Parameter	Results obtained with and without N_2 _sparging at *D *(h^-1^) of:		
	
	0.05 (100 mL/min N_2_)	0.15 (100 mL/min N_2_)	0.05 no stripping
NADH/NAD (mol/mol)	0.13 ± 0.02	0.10 ± 0.00	0.12 ± 0.00
*q_NADH _*produced (mmol/g/h)	4.90 ± 0.2	9.55 ± 0.03	5.8 ± 0.7
*q_NADH _*used (mmol/g/h)	0.44 ± 0.03	2.6 ± 0.5	4.0 ± 0.7
*q_NADH _*produced/*q_NADH _*used (mol/mol)	11	3.7	1.4
NADH available for H_2_:ase (mmol/g/h)	4.5	7.0	1.8

**Figure 3:**
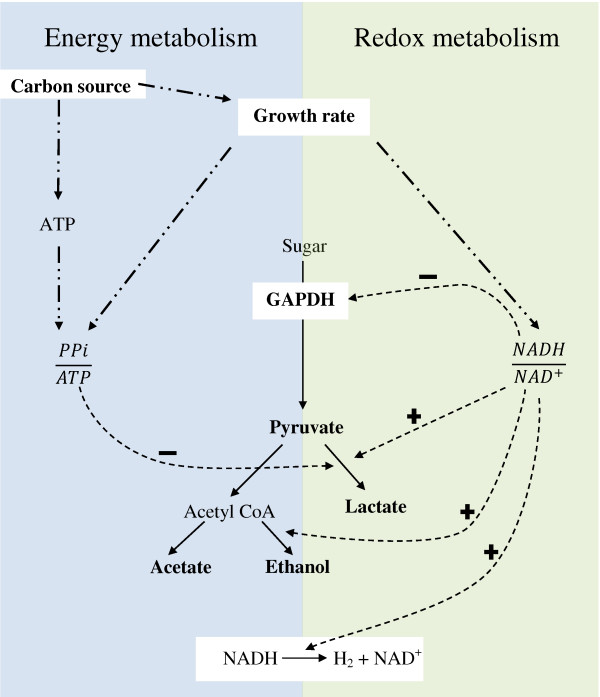
**Summary of the various factors affecting H_2 _tolerance in *C. saccharolyticus*.** Regulation at enzyme level is depicted by the 'simple dashed' line and at metabolic level by 'dash-dotted' line; 'plus' sign shows activation and 'minus' sign shows inhibition.

Under given conditions, the overall glycolytic flux can be measured as pyruvate flux. Hence, it could be argued that, glycolytic flux is a function of the activity of GAPDH (q_pyruvate _= f(v_GAPDH_). The activity of GAPDH, in turn, is a function of NADH/NAD ratio (v_GAPDH _= f(NADH/NAD); Figure 2C; Figure 3). Consistently, a slight decrease in the NADH/NAD ratio appears to result in 94% higher pyruvate flux as a consequence of 9% increase in the GAPDH activity (at D = 0.15h^-1^; Table 1, 2; Figure 2C).

At high *P_H2_*, 53% of the pyruvate flux was redirected to lactate and ethanol (Table 1). This redirection is clearly illustrated by the estimated ratio of the NADH production flux over the NADH flux to reduced products (*q*_NADH_produced/*q*_NADH_used), which is eight folds lower at high *P*_H2 _(Table 3). At this higher flux to reduced products, the NADH available for hydrogen formation is decreased (2.5 folds) as a strategy of the cell to maintain low NADH/NAD ratios at all the conditions, including where the elevated dissolved hydrogen concentration inhibits the NADH-dependent hydrogenase reaction. This inhibition of hydrogen formation is evaluated by thermodynamic analysis.

### Thermodynamic analysis

The critical *P*_H2 _is dependent on the NAD/NADH ratio and the temperature according to (Eq. 7, [39,40]):

(7) $$P_{\text{H}2}=\frac{\left[NAD\left(P\right)H\right]}{NAD{\left(P\right)}^{+}}e{-}^{\left[\frac{{E^{\prime }}^{{}^{0}}NAD{\left(P\right)}^{+}-E^{\prime^{0}}H_{2}}{\frac{RT}{2F}}\right]}$$

where *E*° is the midpoint reduction potential, F is the Faraday's constant, *R *is the ideal gas constant and T is the absolute temperature (K).

Therefore, lower NADH/NAD ratios make the hydrogenase reaction more energetically favourable, which was confirmed experimentally by Veit et al [39]. Consistently, the ethanol-adapted *T. thermohydrosulfuricus *strain, which is more tolerant to H_2 _than the wild-type strain, possessed a GAPDH which could tolerate approximately twice the amount of NADH concentrations as the GAPDH of its wild-type variant [35].

At NADH/NAD ratios of 0.12 mol/mol (Table 3) measured herein, the critical *P*_H2 _for hydrogen production at 70°C should be 12 Pa in the headspace (Eq. 7) and thus H_2_-generation should not be possible to occur spontaneously at 67 kPa. Yet, *C. saccharolyticus *cultures were able to produce H_2 _at this high *P*_H2_.

One way to circumvent this apparent contradiction could be via the substrate specificity of different hydrogenase enzymes. Based on sequence similarity, *C. saccharolyticus *possesses two distinct hydrogenases, one NADH-dependent Fe-only hydrogenase (Csac_1860-1864) and one ferredoxin (Fd)-dependent, membrane-associated NiFe-hydrogenase (Csac_1540-1545; [17]). Given that the redox potential of the Fd couple (Fd_red_/Fd_ox_) is close to that of H_2 _(approx. -400 mV, albeit depending on the involved enzyme [41]), the reaction is energetically favourable even at *P*_H2 _close to 39 kPa. Therefore, it is possible that the NiFe hydrogenase-catalyzed reaction in *C. saccharolyticus *is still functioning even at elevated dissolved H_2 _concentrations. It is noteworthy that the NADH-dependent Fe-only hydrogenase in *T. tengcongensis *was down regulated at high *P*_H2_, while the Fd-dependent hydrogenases were constitutively expressed, irrespective of the *P*_H2 _[42].

An alternative explanation can be proposed related to the finding of Schut and Adams [43], concerning the Fe-only hydrogenase in *T. maritima *using NADH and Fd_red _simultaneously in a bifurcating manner. This novel bifurcating hydrogenase could therefore catalyze the unfavourable oxidation of NADH to H_2 _by using the exothermic oxidation of Fd_red _to drive the reaction. It is noteworthy that the sequence of the Fe-only hydrogenase in *C. saccharolyticus *(Csac_1860-1864) is similar to the bifurcating hydrogenase in *T. maritima *(TM1424-TM1426 [43]). However, it remains to be investigated whether this hydrogenase enzyme in *C. saccharolyticus *possesses a bifurcating function.

### Energy metabolism and its impact on lactate and hydrogen formation

The total ATP and PPi pool in *C. saccharolyticus *increased with the growth rate (Table 4), which is in contrast to what was reported for ATP for *C. cellulolyticum *[32]. In addition, increased levels of both ATP and PPi were observed in the absence of sparging (Table 4) probably due to cell lysis caused by high dissolved CO_2 _concentrations as ATP and PPi were released in the culture broth [15,31]. The latter was confirmed by lower biomass concentration (Table 1). The levels of ATP and PPi are in the same range as under stationary growth of *C. saccharolyticus *[24] and the PPi/ATP ratios are low in all conditions (Table 4). This suggests that LDH should be present in an active configuration during all conditions. However, due to the low NADH/NAD ratio of 0.1 mol/mol, the sensitivity of LDH to changes in PPi/ATP ratio is stronger [24]. In addition, the level of LDH depends on the cultivation condition (Table 2). Consequently, the slightly higher PPi/ATP ratio reduces the estimated activity of LDH 10 and 2.5 folds at low *D *and sparged conditions compared to the non-sparged and high *D *condition, respectively (Table 2, LDHv), partly explaining the significantly lower lactate yields in these conditions. However, the overall glycolytic and shifts in by-product formation is complex and merits more in depth studies. In addition, there is a competition for pyruvate at the pyruvate node (Table 1). Therefore, LDH kinetics alone could not explain the insignificant amount of lactate formed at higher *D *(0.15 h^-1^) even at higher measured LDH activity in this condition (Table 1 and 4).

**Table 4:** ATP and PPi levels at steady states of different dilution rates of *C.saccharolyticus *cultures in the presence and absence of N_2 _sparging

Parameter	Results obtained with or without stripping and at D (h^-1^) of:		
	
	0.05 (100 mL/min N_2_)	0.15 (100 mL/min N_2_)	0.05 no stripping
ATP mM	0.67 ± 0.07	0.80 ± 0.06	2.0 ± 0.2
ATP μmoles/g of cells	3.06 ± 0.32	3.66 ± 0.26	8.95 ± 0.91
PPi mM	0.92 ± 0.05	0.81 ± 0.07	2.1 ± 0.4
PPi μmoles/g of cells	4.20 ± 0.22	3.69 ± 0.34	9.62 ± 1.69
PPi/ATP	1.37	1.01	1.05

### Effect of the carbon source on H_2 _tolerance

The H_2 _tolerance in *C. saccharolyticus *is not only dependent on the growth phase of the organism (Figure 1). The results obtained in this study indicated that the critical *P*_H2 _for initiating lactate formation of *C. saccharolyticus *when grown on glucose was significantly higher than that of previously reported when the organism was grown on sucrose [23]. This led us to investigate whether various carbon sources would allow *C. saccharolyticus *to possess different H_2 _tolerances in an experimental set-up similar to that used by van Niel et al [23]. The fermentations were carried out in batch mode and the gas outlet of the bioreactor was closed at the beginning of the lag phase, leading to a build-up of the total pressure in the vessel due to accumulation of H_2 _and CO_2_. Indeed, the acetate and lactate fluxes were considerably influenced by the carbon source. For instance, the acetate/lactate ratio was 6 in the experiments on xylose, which can be compared to the significantly lower acetate/lactate ratio of 0.26 previously observed on sucrose [23]. The acetate and the lactate yield were 1.2 and 0.21 mol/mol C6, respectively on xylose. In addition, lactate formation remained low in cultures on xylose, during the entire time span and acetate production still continued at *P*_H2 _up to 60 kPa (data not shown), while lactate was formed on sucrose when H_2 _accumulated beyond 10-20 kPa [23]. Every mol of lactate formed deprives the cells not only of a mol of H_2 _but also from obtaining an extra ATP. This is in accordance with previous work showing how the glycolytic flux is significantly increased when xylose is used as carbon source compared to that of sucrose [44]. High throughput technologies can be used to investigate this further in the future. Thus, the observed variability in the extent and sensitivity of lactate formation is also related to the energy metabolism of the cells that may vary with each carbon source (Figure 3; [11]).

## Conclusions

*C. saccharolyticus *has the attractive property of producing high H_2 _yields under ideal conditions. When the *P*_H2 _rises it has the ability to maintain glycolytic flux by regulating GAPDH. Required GAPDH activity is attained by keeping the NADH/NAD ratio relatively low through redistributing its metabolism towards more reduced end products, including lactate and ethanol. The results herein reveal that these redistributions are not solely dependent on the *P*_H2_, but also on the growth state of the organism and the carbon source fermented. Although ethanol is produced, lactate remains the main alternative for *C. saccharolyticus *for reoxidizing NADH. For an economically attractive industrial application of *C. saccharolyticus*, hydrogen yields need to be kept maximized, for which metabolic shift to lactate should be kept at bay. In addition, H_2 _production should be achieved preferably without the need for sparging gas to prevent central costs for the gas-upgrading process [45]. A critical *P*_H2 _should be set so as not to adversely affect the growth rate or biomass yield of the organism or to enhance lactate formation. This should be combined with a careful selection of the feedstock, based on the type of substrates, and operating at adequately low osmotic pressures [15]. Thus, according to our results, a xylose-rich feedstock is preferred over a sucrose-rich one, since the latter enforces an earlier effect of *P*_H2 _on growth and lactate formation than the former. If H_2 _production is possible on a xylose-rich lignocellulosic feedstock and accomplished at high yields at high *P*_H2_, it is definitely a critical step further towards a cost-effective biohydrogen process.

## Abbreviations

*D*: dilution rate; h^-1^; *q*_acetate_: specific formation/consumption rate;mmol (gCDW)^-1 ^h^-1^; *q*_ethanol_: specific formation rate of ethanol; mmol (gCDW)^-1 ^h^-1^; *q*_lactate_: specific formation rate of lactate; mmol (gCDW)^-1 ^h^-1^; *q*_NADH_: produced specific formation rate of NADH; mmol (gCDW)^-1 ^h^-1^; *q*_NADH_: used specific formation rate of NADH used for lactate and ethanol; mmol (gCDW)^-1 ^h^-1^; *q*_glucose_: specific consumption rate of glucose; mmol (gCDW)^-1 ^h^-1^; *q*_pyruvate_: specific formation rate of intracellular pyruvate; mmol (gCDW)^-1 ^h^-1^; *Y*_x/ATP_: energetic biomass yield; gCDW molATP^-1^; *μ*: specific growth rate; h^-1^; *P*_H2_: partial H_2 _pressure; kPa; *K*_i_: inhibition constant

## Competing interests

The authors declare that they have no competing interests.

## Authors' contributions

KW planned the content of the article. KW also planned and preformed the batch experiments and was involved in the planning of the continuous cultures experiments and enzyme kinetics experiments. SP planned and performed the continuous cultures, enzyme kinetics and metabolite analysis. KW and SP both wrote a part of the paper. EvN was involved in the planning of the experiments and supervised the processes. EvN also critically reviewed the text. All authors have read and approved the manuscript.
